# *GRM4* gene polymorphism is associated with susceptibility and prognosis of osteosarcoma in a Chinese Han population

**DOI:** 10.1007/s12032-014-0050-4

**Published:** 2014-06-11

**Authors:** Chaoyin Jiang, Hua Chen, Lei Shao, Yang Dong

**Affiliations:** Department of Orthopedics, Shanghai Jiao Tong University Affiliated Sixth People’s Hospital, Shanghai, 200233 China

**Keywords:** Osteosarcoma, Single-nucleotide polymorphisms, Susceptibility, *GRM4*

## Abstract

Osteosarcoma (OS), the most common primary bone malignancy, occurs primarily in adolescents and young adults. In earlier genome-wide association studies, rs7591996, rs10208273, rs17206779 and rs1906953 were identified as candidate loci for OS in Caucasians but the association of these single-nucleotide polymorphisms (SNPs) with OS in a Chinese Han population remains unknown. We measured the frequency of these four variants in a Chinese Han population to better understand the genetic etiology of OS. Polymerase chain reaction sequencing was used to detect the genotypes of four candidate SNPs in peripheral blood samples collected from 168 OS patients and 216 healthy controls. Logistic regression models were used to estimate the odds ratios and 95 % confidence intervals. We found rs1906953 in the glutamate receptor metabotropic 4 (*GRM4*) gene was associated significantly with OS in our Chinese Han population; as with the other SNPs, however, no statistically significant difference was detected. Further analysis showed the association between rs1906953 and OS was independent of gender and age. The rs1906953 locus was not associated with Enneking stages or tumor location; however, it was associated significantly with OS metastasis and prognosis. The *GRM4* gene polymorphism was associated with the susceptibility and metastasis of OS in a Chinese Han population.

## Introduction

Osteosarcoma (OS) is the most common primary malignant bone tumor in children and young adults with a peak incidence in adolescence and a second smaller peak after age 50, affecting approximately four people per million annually in the USA [1, 2]. The metaphyseal regions of the long bones, including the distal femur, proximal tibia and proximal humerus, which are capable of rapid growth, are most likely to be affected [3]. Primary OS arises from primitive mesenchymal cells producing osteoid and later extends to the pulmonary region forming secondary tumors in 10–20 % of patients [4, 5]. Although neoadjuvant chemotherapy and surgical techniques have improved the 5-year survival of OS patients from 20–30 to 70 %, the level of OS treatment has not obtained a significant improvement in recent years and 30 % of newly diagnosed patients eventually died of lung metastasis [6–9]. There have been extensive studies of OS genetics, biology, pathology and clinical aspects in recent years but there has been little progress in our understanding of its pathogenesis and therapeutic effect [10]. Therefore, it is important to study OS-related risk factors and to identify the underlying mechanism in order to be of great benefit to the reduction of morbidity and mortality of the disease.

No predisposing factor is known to be related to OS. The combined effect of genetic and environmental factors has an important role in the pathogenesis of OS. Genome-wide linkage analysis has been extremely limited in studies of polygenic diseases such as OS; so, using single-nucleotide polymorphism (SNP) as a molecular genetic marker is expected to predict disease susceptibility to guide individual treatment. Earlier studies identified many putative genetic susceptibility variants for increasing OS risk in diverse biologically plausible pathways [11–22], including growth and DNA repair; however, the statistical power of these studies has been limited by small sample sizes [23]. Data regarding the role of common genetic variation in OS risk are also sparse.

Methods for the analysis of genome-wide association studies (GWAS) have been promoted greatly by the HapMap Project data [24]. GWAS are an examination of genome-wide common genetic variants in different individuals to find disease or quantitative trait-related variants. Recently, GWAS in large samples of Caucasians recruited by Savage et al. [25] identified two novel susceptibility loci for OS. In the combined samples, the two novel SNPs (rs1906953 and rs7591996) showed unequivocal evidence of association with OS, with a genome-wide significance threshold of *P* = 5 × 10^−8^. Moreover, the other SNPs (rs10208273 and rs17206779) nearly met the genome-wide significance level for association with OS (*P* = 2.9 × 10^−7^ and 5.1 × 10^−7^). The above four SNPs are associated with OS in Caucasians, but whether they are associated with the incidence of OS in a Chinese Han population has not been estimated. We investigated the association between rs1906953, rs7591996, rs10208273 and rs17206779 polymorphisms and OS susceptibility in a Chinese Han population.

## Materials and methods

### Study design and population

OS cases (*n* = 168) in this study were recruited from the Sixth Affiliated Hospital of Shanghai Jiao Tong University School of Medicine, and all were diagnosed by postoperative histopathological examination. The 216 cancer-free control subjects were chosen from healthy physical examinees. Blood samples of subjects were collected at orthopedic surgery departments between 2008 and 2012, and detailed information about tumor location, stage and histological type of OS was obtained from medical records. All patients were followed up regularly (every 24 months); their survival time, date of death and last follow-up time were recorded. All individuals were self-described as Chinese Han. The institutional review boards of Shanghai Jiao Tong University approved the protocol for this study, and written consent was obtained from all subjects.

### Genomic DNA extraction and genotyping assays

Genomic DNA was extracted from blood specimens using a Fujifilm QuickGene-610L system according to the manufacturer’s protocol. A spectrophotometer was used to measure the concentration of DNA, which was then stored at −20 °C. Sequence information for four candidate SNPs (rs7591996, rs10208273, rs17206779 and rs1906953) was obtained from the National Center for Biotechnology Information (NCBI) SNP database (http://www.ncbi.nlm.nih.gov/snp/). Sequences containing the four candidate SNPs were amplified by polymerase chain reaction (PCR) using high-fidelity Taq polymerase (Roche) with appropriate primers (Table 1). All PCR products were purified and sequenced with an ABI 377 DNA Sequencer. Genotype analysis was done with GENESCANTM and GENOTYPERTM software. Genotype results were recorded and entered into the computer separately by two researchers.

**Table 1:** Primers used for PCR sequencing of genotypes

Gene	Primers	Sequence
rs7591996	Forward	5′-TGCCCTGGACCATATGCATT-3′
	Reverse	5′-GCATCCCAGAAAGAAAGCCC-3′
rs10208273	Forward	5′-AAGGCAATTTTCACAGAGCC-3′
	Reverse	5′-TGATGCCGAAGACAACTTGA-3′
rs17206779	Forward	5′-TCTTCCTCTGGGTGGCTCTC-3′
	Reverse	5′-CAAGGTGGATTGGCCCAA-3′
rs1906953	Forward	5′-CTTGCAGCACTTCCCCAT-3′
	Reverse	5′-TTCCTTGTCTGGAGCCCAGT-3′

### Statistical analysis

Pearson’s *χ* ^2^ test was used to detect whether the genotype frequencies deviated from the Hardy–Weinberg equilibrium (HWE) in the experimental and control groups for each SNP. Logistic regression models were used to estimate the odds ratio (OR) and 95 % confidence intervals (CI) for the strength of the association with OS risk, respectively. The association between rs1906953 genotype frequencies and clinicopathological characteristics of OS was calculated using unconditional logistic regression and then adjusted for age, gender and body mass index (BMI). The Kaplan–Meier method was used to estimate the probability of disease-free survival. Log-rank analysis was used to assess the significance of the differences among OS patients with three genotypes of rs1906953 (TT, TC and CC). Statistical analysis was done with SPSS version19.0 statistical software. Differences with *P* ≤ 0.05 were considered statistically significant.

## Results

### Clinical features of the study subjects

The clinical features of the 384 subjects are given in Table 2. Among the 168 OS patients, 41.67 % (70/168) were female and 58.33 % (98/168) were male. The median age of the OS cases was 20.41 years (range 9–75 years) and 20.58 years (range 10–78 years) for the controls. The average BMI was 22.07 (±1.87) kg/m^2^ for the OS patients and 21.89 (±1.86) kg/m^2^ for the controls. Differences of gender, age and BMI were not statistically significant between the case and control groups (*P* = 0.462, 0.572 and 0.262, respectively). All participants were Chinese Han.

**Table 2:** Comparison of clinical features between the case and control groups

Variable	Case	Control	*P* value
No. (*n*)	168	216	
Female [*n* (%)]	70 (41.67)	82 (37.96)	0.462
Age (years)	20.09 ± 9.32	20.53 ± 11.79	0.737
BMI (kg/m^2^)	22.07 ± 1.87	21.89 ± 1.86	0.262
Tumor metastasis [*n* (%)]	34 (20.24)		
Enneking stages [*n* (%)]			
I	25 (14.88)		
II	120 (71.43)		
III	23 (13.69)		
Location [*n* (%)]			
Trunk	17 (10.12)		
Limbs	151 (89.88)		

### Candidate SNPs associated with OS

The four candidate SNPs (rs7591996, rs10208273, rs17206779 and rs1906953) and incidence risk of OS are given in Table 3. Genotype distributions of rs7591996, rs10208273, rs17206779 and rs1906953 were consistent with HWE in the control group (*P* = 0.898, 0.689, 0.372 and 0.700, respectively) and in the OS group (*P* = 0.965, 0.354, 0.537 and 0.763, respectively). As with rs7591996, rs10208273 and rs17206779 allele frequency distributions, no statistically significant difference (*P* = 0.608, 0.639 and 0.548, respectively) was observed between the case and control groups (Table 3). However, with respect to the rs1906953 allele frequency distribution, a significant difference of *P* = 0.002 was observed between the case and control groups. The association analysis showed patients carrying the T allele had a higher risk of OS (OR 1.57; 95 % CI 1.18–2.09) compared to the C allele (Table 2).

**Table 3:** Summary of association results of four SNPs between 168 cases and 216 controls

SNP Genotype	Case (*n* = 168)	Control (*n* = 216)	OR (95 % CI)	*P* value
rs7591996				
AA	15 (8.93)	21 (9.72)		0.870
AC	70 (41.67)	94 (43.52)		
CC	83 (49.40)	101 (46.76)		
A	100 (29.76)	136 (31.48)	1.08 (1.48–0.8)	0.608
C	236 (70.24)	296 (68.52)		
rs10208273				
AA	28 (16.67)	31 (14.35)		0.823
AG	74 (44.05)	98 (45.37)		
GG	66 (39.29)	87 (40.28)		
A	130 (38.69)	160 (37.04)	1.07 (1.44–0.8)	0.639
G	206 (61.31)	272 (62.96)		
rs17206779				
TT	24 (14.29)	35 (16.2)		0.837
TC	74 (44.05)	96 (44.44)		
CC	70 (41.67)	85 (39.35)		
T	122 (36.31)	166 (38.43)	1.09 (1.47–0.82)	0.548
C	214 (63.69)	266 (61.57)		
rs1906953				
TT	45 (26.79)	36 (16.67)		0.010
TC	82 (48.81)	101 (46.76)		
CC	41 (24.40)	79 (36.57)		
T	172 (51.19)	173 (40.05)	1.57 (2.09–1.18)	0.002
C	164 (48.81)	259 (59.95)		

### Association between rs1906953 genotype frequency and clinicopathological features in OS patients

A stratified analysis of clinicopathological characteristics of OS was used to further investigate the relationship between rs1906953 and the incidence of OS. The results are given in Table 4. The genotype TT frequency of rs1906953 in tumor metastasis patients (44.12 %) was greater compared to patients without tumor metastasis (26.67 %), and the difference in frequency distribution between genotypes reached significance (*P* = 0.036). No significant difference was observed with respect to sex, age, Enneking stage or tumor location and the rs1906953 genotype.

**Table 4:** Association between genotype frequencies of rs1906953 and clinicopathological features in OS patients

Variable	*n*	TT	TC	CC	*P* value
Gender					
Female	70	19 (27.14)	33 (47.14)	18 (25.71)	0.924
Male	98	26 (26.53)	49 (50.00)	23 (23.47)	
Age					
>20	64	15 (23.44)	31 (48.44)	18 (28.13)	0.600
≤20	104	30 (28.85)	51 (49.04)	23 (22.12)	
Tumor metastasis					
Yes	34	15 (44.12)	12 (35.29)	7 (20.59)	0.036
No	134	30 (22.39)	70 (52.24)	34 (25.37)	
Enneking stage					
I	25	7 (28.00)	12 (48.00)	6 (24.00)	0.696
II	120	32 (26.67)	61 (50.83)	27 (22.50)	
III	23	6 (26.09)	9 (39.13)	8 (34.78)	
Location					
Trunk	17	5 (29.41)	7 (41.18)	5 (29.41)	0.830
Limbs	151	40 (26.49)	75 (49.67)	36 (23.84)	

### Association between the rs1906953 genotype and prognosis of patients with OS

**Fig. 1:**
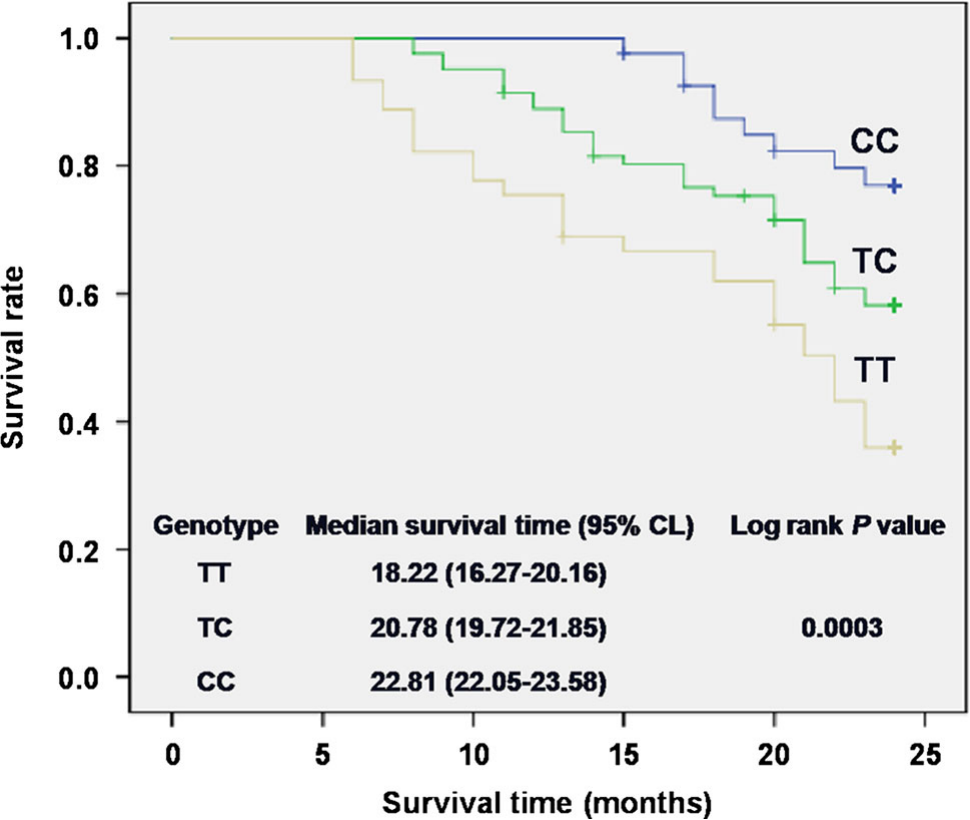
Analysis for effects of the rs1906953 genotype on survival of the OS group

Survival analysis showed that for rs1906953, OS patients carrying the TT genotype are likely to be correlated with a poor survival rate (*P* = 0.0003 by Log-rank analysis; Fig. 1). Patients carrying the TT genotype had a median survival time of 18.22 months, and patients with the TC genotype (20.78 months) and CC genotype (22.81 months) had the best outcome.

## Discussion

Genetic and environmental factors both contribute to the cause of OS, and genetic background has the most important role. The finding of more and more susceptibility loci and genes related to OS in recent years will provide important insight into the diagnosis of the disease and aid the design of new preventive and therapeutic strategies for OS patients. Importantly, we confirmed the earlier finding that rs1906953 in the glutamate receptor metabotropic 4 (*GRM4*) gene is associated with OS in the Chinese Han population through the association analysis, which once more provides strong evidence for this locus.

We showed for the first time that there is an association between rs1906953, located in intron 7 of *GRM4* at 6p21.3, and OS in a Chinese Han population. Our results showed the allele T frequency of the OS case group (51.19 %) was greater compared to the control group (40.05 %) and those carrying the TT genotype had a higher risk of OS. *GRM4* is a family of G protein-coupled receptors that encode the group III metabotropic glutamate receptor 4 (mGluR4), and its role in intracellular signaling and inhibition of the cyclic AMP (cAMP)-signaling cascade has been reported [26, 27].

The important role of glutamate in intercellular communication in the central nervous system (CNS) has been extended in recent years to non-neural systems [28–30]. The glutamate-signaling system has been shown to be essential in bone remodeling and homeostasis [29, 31, 32]. The disrupted normal glutamate signaling aggravates several bone diseases. Numerous cancers, including breast, prostate and lung, that can metastasize to bone have been shown to express several glutamate receptors and transporters [33, 34]. Human OS cell lines expressing several glutamate receptors are known to be associated with proper tumor cell function because inhibited glutamate receptors lead to limited cell growth and pharmacologic prevention of glutamate release that results in blocked differentiation and increased levels of apoptosis [35]. The important role of the cAMP pathway in OS has been demonstrated in mice, in which a cAMP-dependent protein kinase (Prkar1a) has been shown to suppress OS tumor growth [36, 37]. Together, these results suggest *GRM4* is implicated in OS.

The reference SNP (rs1906953) is located in intron 7 of *GRM4*, which is expressed abundantly in nerve tissue (including brain, spinal cord and retina) and relatively little in other tissues. To further test whether the OS-associated SNPs regulate the expression of *GRM4*, we inspected three *cis*-gene expression quantitative trait loci (*cis*-eQTL) databases of European Caucasian populations [38–40] and found rs1906953 was not correlated with the expression of *GRM4* or other genes. The index SNP, rs1906953, maps to a DNase I hypersensitive region based on the Encyclopedia of DNA Elements (ENCODE) data set, raising the distinct likelihood that the variant is within a region accessible to open chromatin and could manifest active regulatory elements [41].

Several studies have suggested OS has a high incidence in young children and males of all ages and is often located in long tubular bones [42]. The results of some studies suggested being taller than average and with a greater birth weight at diagnosis are associated with increased OS risk [43–45]. Therefore, we analyzed the association of rs1906953 with some clinical parameters, including gender, age, location and distant metastasis. The genotype of rs1906953 is associated with the susceptibility of OS in our study, but not specifically to cases with different gender, age and location of the tumor. This indicates *GRM4* is involved in signaling pathways that stimulate the formation of OS independent of gender, age and location.

Distant metastasis association analysis showed the genotype of rs1906953_TT was correlated significantly with metastasis of OS. Survival analysis showed for rs1906953 that the median survival time of OS patients with the TT genotype was significantly shorter compared to the TC and CC genotypes. This result is consistent with several earlier findings that *GRM4* expressed in human OS cells is associated with its proper function and its expression in pediatric CNS tumors, rhabdomyosarcoma, multiple myeloma and colorectal cancer is predicted with poor prognosis [35, 46–49]. Therefore, molecular biological studies are needed to confirm these two findings. On the basis of these results, we suggest *GRM4*-related signaling is involved in the metastasis of OS and affects the survival of OS patients; however, more studies of the underlying molecular and biological mechanisms are needed to further confirm these results.

In summary, we showed the *GRM4* gene polymorphism rs1906953 is associated with the incidence of OS and with the metastasis and survival of OS patients in a Chinese Han population. There are some limitations in our case–control study. Because all patient cases in this study were recruited from one hospital, it is difficult to avoid the inherent selection bias. In addition, because of the low incidence of OS, the number of OS patients in the study is small, which makes the statistical power of our results low. Therefore, studies with greater numbers of patients are needed to be confident that the *GRM4* gene polymorphism rs1906953 is a genetic marker in predicting the incidence and prognosis of OS in a Chinese Han population.
